# Curatively Resected Mesenteric Lymph Node Recurrence of Ewing Sarcoma

**DOI:** 10.70352/scrj.cr.25-0386

**Published:** 2025-08-19

**Authors:** Ayaka Tachikawa, Kazushige Kawai, Akira Dejima, Sakiko Nakamori, Hiroki Kato, Soichiro Natsume, Misato Takao, Hiroshi Shiratori, Daisuke Nakano, Toru Motoi, Toshihide Hirai

**Affiliations:** 1 Department of Colorectal Surgery, Tokyo Metropolitan Cancer and Infectious Diseases Center Komagome Hospital, Tokyo, Japan; 2 Department of Pathology, Tokyo Metropolitan Cancer and Infectious Diseases Center Komagome Hospital, Tokyo, Japan; 3 Department of Musculoskeletal Oncology, Tokyo Metropolitan Cancer and Infectious Diseases Center Komagome Hospital, Tokyo, Japan

**Keywords:** Ewing’s sarcoma, mesenteric lymph node, metastasis

## Abstract

**INTRODUCTION:**

We report herein a rare case of Ewing sarcoma that metastasized to the mesenteric lymph nodes.

**CASE PRESENTATION:**

The patient was a 40-year-old female with Ewing sarcoma of the 1st lumbar vertebra, which was treated with chemotherapy and stereotactic radiotherapy. No local recurrence or distant metastasis was observed during the first 3-year follow-up period after treatment. Three years later, she presented to the emergency department with muscle weakness. A 60-mm lesion in the right parieto-occipital lobe of the brain and a 40-mm tumor in the small bowel mesentery were detected. Emergency craniotomy confirmed a cerebral metastasis of the Ewing sarcoma. The patient subsequently underwent 6 cycles of ifosfamide (IFM) monotherapy, which reduced the mesenteric tumor to 10 mm in size. Surgical resection was performed with clear margins. Histopathological examination of the mesenteric lymph nodes confirmed the findings of the emergency craniotomy. The patient continues to receive IFM monotherapy as adjuvant chemotherapy. Although brain metastases developed at postoperative months 1, 6, and 10, no intra-abdominal recurrence was observed during the 1-year surveillance period.

**CONCLUSIONS:**

While Ewing sarcoma can metastasize to isolated distant lymph nodes, oligometastases can be treated with surgical resection.

## Abbreviations

3-yr EFS
: 3-year event-free survival
ES
: Ewing sarcoma
EURO-E.W.I.N.G.
: the European Ewing Tumor Working Initiative of National Groups
IFM
: ifosfamide
VDC/IE
: vincristine, doxorubicin, cyclophosphamide/ifosfamide, and etoposide

## INTRODUCTION

ES is a type of undifferentiated small, round cell sarcoma. Although this tumor primarily arises in bone, about 12% of cases are extraskeletal. ES is the second most common bone tumor in adolescents and young adults, and is slightly more common among males.^1–3)^ Lymph node metastases are rarer and, in most cases, are associated with disseminated disease.^4)^ Advances in treatment have improved the 5-year survival rate of patients with a localized lesion to nearly 80%. However, the 5-year survival rate in patients with a recurrence, for which there is no standard treatment, remains less than 30%.^1,5,6)^

We describe herein a rare case of a recurrent, mesenteric lymph node metastasis of ES originating in a vertebra. The early occurrence of oligometastases in a lymph node as the initial site of recurrence, concomitant with a hematogenous brain metastasis, is rare. In the present case, the patient achieved remission after receiving a combination of chemotherapy and surgical resection.

## CASE PRESENTATION

**Fig. 1:**
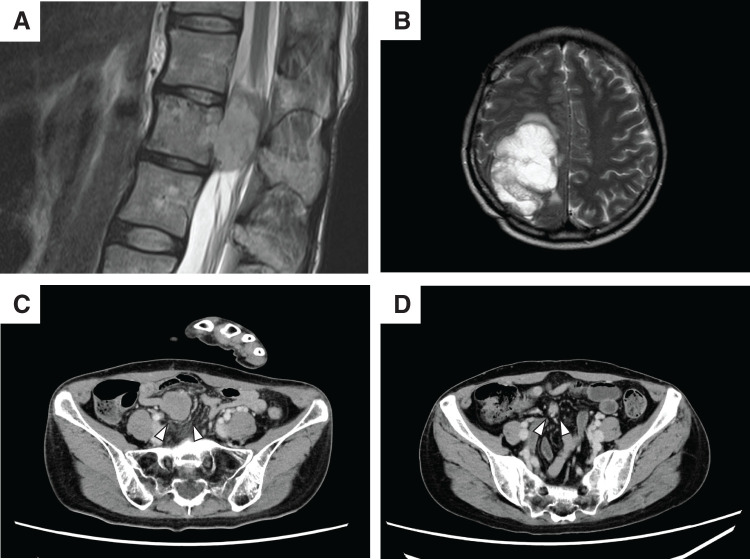
Imaging studies. (**A**) T2-weighted MRI demonstrating an osteolytic lesion in the 1st lumbar vertebra. (**B**) T2-weighted MRI demonstrating a metastasis with high signal intensity in the right parieto-occipital lobe, which developed 3 years after treatment of the primary lesion. (**C**) Contrast-enhanced abdominal CT showing mesenteric lymph node metastasis (white arrowheads), which developed simultaneously with the brain metastasis. (**D**) The mesenteric metastasis (white arrowheads) markedly shrank (to 30 mm in size) after 6 cycles of IFM therapy. IFM, ifosfamide

A 40-year-old female patient presented with lower back pain and lower limb numbness. T2-weighted MRI revealed an osteolytic lesion in the 1st lumbar vertebra (**Fig. 1A**), which was diagnosed as ES on the basis of bone biopsy findings. The patient received 7 courses of VDC/IE chemotherapy (vincristine, doxorubicin, cyclophosphamide/IFM, and etoposide) and stereotactic radiotherapy with curative intent (50 Gy in 5 fractions). The follow-up strategy after the initial treatment consisted of monthly outpatient visits, chest-to-abdomen CT and MRI for the spine every 6 months, and full-body PET-CT once a year. No imaging tests were performed for possible brain metastases. No local recurrence or distant metastasis was observed during the post-treatment follow-up period, during which chemotherapy was not administered.

Three years after completing the initial treatment, the patient presented to the emergency department with muscle weakness. T2-weighted MRI revealed a solitary 60-mm lesion in the right parieto-occipital lobe of the brain (**Fig. 1B**). Additionally, a solitary 40-mm lesion was detected within the small bowel mesentery with no visible continuity with the intestinal tract on CT (**Fig. 1C**). Emergency craniotomy and tumor resection were performed for the brain lesion, which was histopathologically diagnosed as a cerebral metastasis of ES. Radiation therapy was administered at postoperative week 3. One week thereafter, high-dose IFM monotherapy was administered in accordance with the UK guidelines for second-line therapy for Ewing sarcoma.^7)^ The decision to administer the treatment was made by a multidisciplinary team. The tumor’s response to the therapy was assessed every 2 months. By the time 6 IFM cycles were completed, the mesenteric tumor had shrunk to 10 mm, enabling surgical resection. Given its responsiveness to chemotherapy, the mesenteric tumor was strongly suspected of being a metastasis of the primary ES. Surgical resection was performed, and intraoperative findings revealed no evidence of peritoneal dissemination. The lesion was palpable as a nodule within the ileal mesentery. The intestinal tract was tumor-free, and no lymph node enlargement was observed in the surrounding area. The mesenteric tumor was resected with clear margins. The operative time was 92 minutes, and there was no remarkable blood loss. The patient had a favorable postoperative course and was discharged without complications on postoperative day 7.

**Fig. 2:**
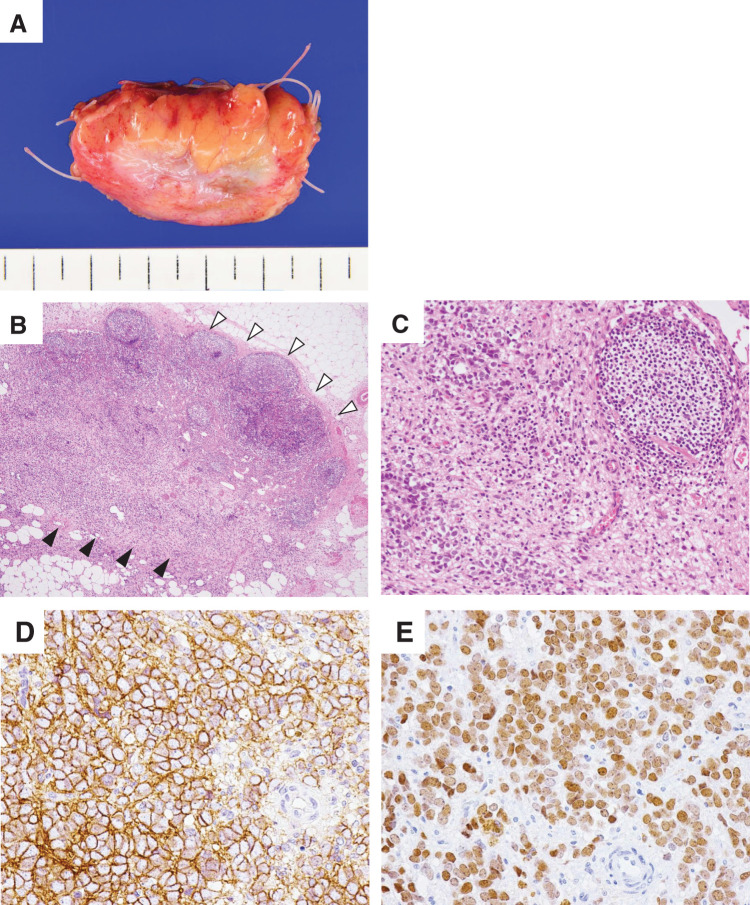
Resected specimen and histopathological findings. (**A**) A mesenteric nodule covered with peritoneum was dissected with a clear margin. (**B**) Tumor cells (black arrowheads) had infiltrated the lymph nodes (white arrowheads, HE staining, low magnification). (**C**) Relatively monomorphic small, round neoplastic cells with hyperchromatic nuclei and scant eosinophilic cytoplasm were observed (HE staining, high magnification). (**D**, **E**) Immunohistochemically, the tumor cells were diffusely positive for CD99 (**D**) and NKX2.2 (**E**). HE, hematoxylin–eosin

The resected specimen had a maximum diameter of 45 mm, a negative surgical margin, and was surrounded by adipose tissue (**Fig. 2A**). Histopathological examination revealed that the tumor involved the mesenteric lymph nodes (**Fig. 2B**), whose structure showed considerable degradation. Diffuse proliferation of small round tumor cells with monotonous, round nuclei, fine nuclear chromatin, and scant eosinophilic cytoplasm was observed (**Fig. 2C**). These findings were consistent with typical ES. Immunohistochemically, the tumor cells were diffusely positive for CD99, a non-specific but traditional immunohistochemical marker for ES. They were also positive for NKX2.2, which has recently begun to be used as a more reliable diagnostic marker than CD99.^8)^ The final pathological diagnosis was metastasis of typical ES to the mesenteric lymph nodes. The patient is continuing to receive IFM monotherapy as adjuvant chemotherapy. Although brain metastases occurred at postoperative months 1, 6, and 10 and were treated with stereotactic radiotherapy, no intra-abdominal recurrence was observed during the 1-year surveillance period.

## DISCUSSION

ES histologically appears as uniformly undifferentiated small round cells, showing a specific fusion between the *EWS* and *ETS* family of genes.^1)^ Common, primary sites are skeletal and include the pelvis (24%), femur (16%), and ribs (12%).^1,3)^ ES rarely occurs in patients older than 30 years of age, with nearly 80% of the patients being younger than 20 years.^1)^ Advances in chemotherapy and the combination of new, multimodal therapies with local therapy^3)^ have improved the 5-year survival rate from 44% in the 1980s to nearly 80% in the 2000s.^4,9)^ However, the 5-year survival rate following a recurrence, for which no standard treatment has yet been established, remains below 30%.^1,5,6)^ ES commonly metastasizes to the lungs and bones but rarely to lymph nodes,^4,10,11)^ and the incidence of lymph node metastases of primary ES is unclear. In their 2007 study, Völker et al. observed a lymph node metastasis in 8 of 46 patients aged 1–18 years (17.4%).^12)^ Lymph node metastases in adults are even rarer and, in most cases, are associated with disseminated disease.^4)^ In contrast, a key finding in the present case was the early occurrence of oligometastases to the lymph nodes, along with hematogenous metastasis to the brain as the initial sites of recurrence.

There is currently no standard treatment for distant metastases of ES. Generally, multimodal therapy combining chemotherapy with local treatments, such as surgery and radiation therapy, is administered.^13)^ A supplementary analysis of a randomized controlled trial conducted by the EURO-E.W.I.N.G. 99 from 1998 to 2006 at the Muenster trial center^11)^ evaluated the impact of local therapy following chemotherapy on the prognosis of extrapulmonary metastases of primary, disseminated, and multifocal ES. Patients receiving a combination of surgery and radiation therapy had a 3-yr EFS rate of 56%, while those who received surgery alone, radiation therapy alone, and no local therapy had a 3-yr EFS rates of 33%, 35%, and 16%, respectively. Furthermore, multivariate analysis demonstrated that the absence of local therapy was an independent factor of poor prognosis. These findings suggest that administering local therapy with chemotherapy is crucial for improving the prognosis of patients with metastases.

In the present case, emergency craniotomy and tumor resection for the cerebral metastasis were first performed in response to the rapid progression of muscle weakness. The patient underwent IFM monotherapy as postoperative chemotherapy for the brain metastasis and preoperative chemotherapy for the mesenteric metastasis. The latter responded well to neoadjuvant chemotherapy, and no intra-abdominal recurrence was observed during postoperative year 1. However, the brain metastasis recurred at months 1, 6, and 10 after the mesenterectomy at sites that differed from that of the initial brain metastasis, suggesting hematogenous spread or dissemination. Difficulty in controlling the brain metastasis was possibly the dominant prognostic factor in this case.

Brain metastases of sarcomas, including ES, generally have a poor prognosis, with a median overall survival of 4.0 months.^14)^ One of the main reasons for this poor outcome is that most chemotherapeutic agents used to treat sarcomas have poor blood–brain barrier penetration, thereby reducing their efficacy against brain metastases.^15,16)^ In addition, brain metastases reportedly occur in about 3%–6% of Ewing sarcoma cases, and most affected individuals already have or have had metastases to other organs, such as the lungs.^17,18)^ Performing brain CT for surveillance may enable earlier detection of brain metastases; however, considering their low frequency, CT may not be suitable as a routine means of conducting surveillance.

## CONCLUSIONS

We reported a rare case of ES that had metastasized to the mesenteric lymph nodes. While ES can metastasize to isolated, distant lymph nodes, oligometastases can be treated effectively by surgical resection.

## DECLARATIONS

### Funding

The authors declare no funding for this study.

### Authors’ contributions

AT drafted the manuscript.

KK contributed to the study supervision, performed the investigation, and critically revised the manuscript for important, intellectual content.

AD, SN, HK, SN, MT, HS, DN, and TH administered the clinical treatments and performed the surgical procedures and perioperative management.

TM made the pathological diagnosis. All the authors have read and approved the final manuscript.

### Availability of data and materials

Not applicable.

### Ethics approval and consent to participate

This work does not require ethical considerations or approval. Informed consent to participate in this study was obtained from the patient.

### Consent for publication

Informed consent for publication of this case report was obtained from the patient.

### Competing interests

There are no conflicts of interest.
